# Different responses of luminal and glandular epithelium during mouse embryo implantation

**DOI:** 10.3389/fvets.2025.1661930

**Published:** 2025-09-24

**Authors:** Wenjing Cui, Xiangrui Guan, Peisen Liu, Qi-Xin Xu, Juan Xie, Yaqi Jiao, Lin Jin, Peng-Chao Wang, Zhenshan Yang

**Affiliations:** 1 College of Veterinary Medicine, Shanxi Agricultural University, Taigu, China; 2 Shenzhen Maternity and Child Healthcare Hospital, Southern Medical University, Shenzhen, Guangdong, China; 3 Chongqing Key Laboratory of Human Embryo Engineering and Precision Medicine, Center for Reproductive Medicine, Chongqing Health Center for Women and Children, Women and Children’s Hospital of Chongqing Medical University, Chongqing, China; 4 College of Marine Life Sciences, Ocean University of China, Qingdao, China; 5 College of Veterinary Medicine, South China Agricultural University, Guangzhou, China

**Keywords:** luminal epithelium, glandular epithelium, embryo implantation, signaling pathway, RNA sequencing

## Abstract

**Introduction:**

Embryo implantation, a crucial process for establishing and maintaining a successful pregnancy, involves the attachment and invasion of the embryo into the endometrium. The glandular epithelium (GE) within endometrial glands secretes multiple factors to support embryonic development, while the luminal epithelium (LE) covering the endometrial surface directly interacts with the embryo and regulates its invasion. This study uses RNA sequencing to examine the different responses of luminal epithelium (LE) and glandular epithelium (GE) during mouse embryo implantation.

**Methods:**

We performed the RNA-seq using the mouse model of delayed and activated implantation to investigate the distinct regulatory mechanisms of LE and GE at 0 h, 3 h, and 6 h after initiating embryo implantation.

**Results:**

Through RNA sequencing and functional enrichment analysis of LE and GE tissues collected at different time points during activation, we revealed significant functional divergence between these two epithelial compartments across implantation stages. LE might predominantly regulate embryo attachment and initial invasion via activation of JAK-STAT, MAPK, and PI3K-Akt signaling pathways. In contrast, GE may exhibit specialized functions supporting embryonic development and maintaining the uterine microenvironment by modulating retinol metabolism, sphingolipid metabolism, and the Notch signaling pathway. Time-series analysis by Mfuzz further uncovered dynamic response patterns in both epithelial layers following progesterone administration. JAK-STAT and MAPK signaling pathways were significantly up-regulated in the LE after 3 h of treatment with estradiol-17β in mice. Retinol metabolism and glutathione metabolism signaling pathway were up-regulated in the GE after being treated with estradiol-17β in mice.

**Conclusions:**

RNA-seq results showed that LE and GE have different responses during mouse embryo implantation. These findings provide novel insights into the molecular mechanisms underlying embryo-endometrial crosstalk, offering valuable implications for developing therapeutic strategies for implantation-related infertility and optimizing assisted reproductive technologies.

## 1 Introduction

Embryo implantation is a pivotal event that marks the beginning of pregnancy, involving the attachment and invasion of the embryo into the uterine wall. This complex, tightly regulated process unfolds in three phases—apposition, adhesion, and invasion (1), governed by a complex regulatory network involving multiple genes, biomolecules, cytokines, and signaling pathways (2, 3). Despite advances in reproductive medicine, the implantation stage remains a major bottleneck in assisted reproductive technologies (ART). Therefore, a deeper understanding of the molecular and cellular mechanisms underlying embryo implantation is vital for improving fertility treatments and pregnancy outcomes (4).

At the heart of successful implantation lies a finely tuned interaction between the embryo and the endometrial lining (5). The endometrium comprises heterogeneous cell populations, including stromal cells, immune cells, endothelial cells, and epithelial cells (6). Among these, epithelial cells divided into luminal epithelium (LE) and glandular epithelium (GE), play crucial roles in mediating embryo-endometrial communication. While LE and GE share similarities as columnar epithelial cells organized within the same histological monolayer, they exhibit distinct differences in cellular morphology, secretory activity, ultrastructural organization, and spatial localization. LE is located at the top of the supporting matrix fibroblasts, whereas GE is embedded within the matrix layer (7, 8). Understanding the distinct roles of these epithelial subtypes is key to unraveling the cellular dynamics of implantation.

The endometrial LE serves as the first maternal tissue to establish physical interactions with the blastocyst (9). Precise regulation of the LE is crucial for a successful pregnancy (10). The LE primarily guides embryonic positioning via surface molecules (e.g., integrin αvβ3, L-selectin ligands) and facilitates adhesion between trophoblast cells and the epithelium (11, 12). Additionally, they secrete chemokines (e.g., CXCL12) and growth factors (e.g., HB-EGF) to stimulate embryonic activation and invasion (13, 14). Under the influence of ovarian hormones, LE undergoes a transformation from tall columnar to cuboidal morphology and loses polarity, a process that marks the receptive phase (9, 15). In contrast, GE functions as the “logistical hub” for embryonic nutrition and signaling. Glandular epithelial cells primarily secrete nutrients and cytokines to support embryo implantation (16). It secretes leukemia inhibitory factor (LIF) and glycoproteins such as MUC1, regulating implantation and supporting embryonic nutrient supply (17, 18). These secretions are essential for maintaining endometrial receptivity (19). However, despite the recognized importance of LE and GE, their mechanistic role in embryo implantation remains significantly underexplored.

This study investigates the distinct responses of luminal and glandular epithelia during embryo implantation to advance our understanding of the interaction mechanisms between embryos and the maternal uterus. By elucidating the roles of signaling molecules in implantation, this work provides novel insights into the molecular regulatory networks governing embryo attachment. Furthermore, uncovering the differential roles of luminal and glandular epithelia in implantation may open new avenues for infertility treatment, optimization of ART, and research on pregnancy-related disorders.

## 2 Materials and methods

### 2.1 Animals

C57BL/6 mice of 8–12 weeks of age were used in this study and purchased from Slack Laboratory Animal Co., LTD (Hunan, China). The mice were housed in an SPF-grade environment with a room temperature of 22 ± 2°C, a humidity of 50 ± 10%, and a photoperiod of 12 h of light/12 h of darkness, and were allowed to ingest food and water ad libitum. The mice were acclimated for 1 week before the experiment. All animals were approved by the Shanxi Agricultural University Institutional Animal Care and Use Committee (SXAU-EAW-2021M.MQ.003012284) and were handled in accordance with the ARRIVE guidelines.

### 2.2 Delayed implantation and activation mouse model

Female mice were mated with fertile male mice of the same strain to induce pregnancy (day 1 was the date of vaginal plugging), and females examined at 8 a.m. the next day for vaginal plugs were labeled as fertile mice. Nine conceived mice were divided into three groups. To induce delayed implantation, pregnant mice were anesthetized with 0.5 mg/g 2,2,2-tribromoethanol (Sigma, T48402) on the morning of day 4 of gestation and then underwent ovariectomy. The mice were injected daily with 1 mg/mouse progesterone to maintain delayed implantation. Embryo implantation was activated by injection of 25 ng/mouse estradiol-17β on the morning of day 7 (20), and the mice were euthanasia by cervical dislocation 0, 3, and 6 h after the injection, and the materials were collected. It showed that 3 and 6 h of injection did not show the implantation sites by Chicago blue (100 μL, Sigma) injection. However, after 12 h of injection, blue implantation sites were shown in the mice’s uterus (Supplementary Figure S1). This indicates that our delayed implantation and activation mouse model was successful.

### 2.3 Luminal and glandular epithelium isolation

**Figure 1:**
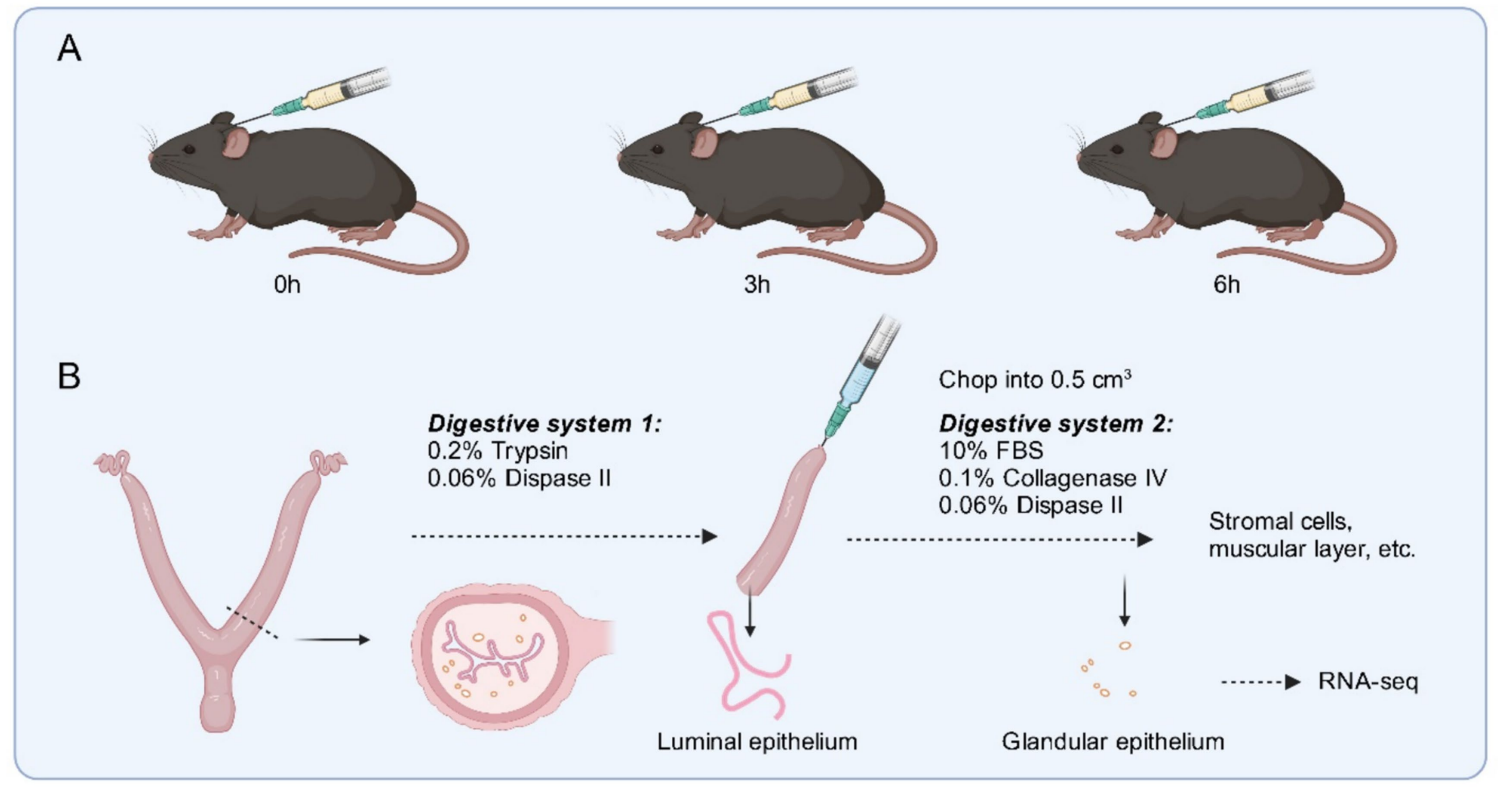
**(A)** Schematic diagram of the delayed and activated implantation model in the study. **(B)** Steps for collecting uterine tissue and isolating the luminal and glandular epithelium. Created with BioRender.com.

The mouse uterus was collected, the uterine horns were removed from the uterine tunica, the uterus was flushed with HBSS (Sigma, SLCB9243), and the flushed fluid was observed under a somatic microscope, with blastocysts to determine mouse pregnancy. The uterus was placed in HBSS digestion solution containing 0.2% trypsin and 0.06% trypsin II (Roche BR, 4942078001) and digested at 4°C for 1 h, and in a water bath at 37°C for 1 h. After completion of the digestion, the epithelium of the uterine lumen was rinsed by drawing HBSS buffer with a 10 mL syringe. The luminal epithelium was washed in HBSS and collected for further study (Figure 1A).

The remaining tissue was sheared into 0.5 cm^3^ pieces and digested in 10% FBS, 0.1% collagenase (Gibco, 17104019), 0.06% dispase II digestion system at 37°C, 810 rpm/30 min in a metal bath, and then 1% DNase (Yeasen, 10607ES15) was added for 5 min. After digestion, the supernatant was passed through a 200-mesh filter. It was washed several times with HBSS medium. The supernatant was collected and passed through a 1,000-mesh filter twice. The final filtrate contained stromal cells; the filtrate was glandular epithelium. The sieve was inverted on a 35 mm petri dish, the retained glandular epithelial cells were backwashed from the sieve membrane, and the precipitate was centrifuged at 1,200 rpm/5 min and collected (Figure 1B).

### 2.4 RNA-seq and data analysis

Total RNA was extracted from LE and GE samples using Trizol RNA reagent (Takara, Dalian, China). RNA concentration and purity were determined by an ND-1000 NanoDrop spectrophotometer (Thermo Fisher Scientific, USA), and an A260/A280 ratio ≥1.8 and A260/A230 ratio ≥2.0 indicated satisfactory RNA quality. RNA integrity was assessed by Agilent 2,100 Bioanalyzer (Agilent Technologies, USA), and only samples with an RNA integrity number (RIN) ≥ 8.0 were used for subsequent library construction. cDNA libraries were generated using the TruSeq RNA Sample Preparation Kit (Illumina, San Diego, CA, USA). RNA sequencing was performed on an Illumina HiSeq 2500 system. Raw data were processed using an in-house computational pipeline, and differentially expressed genes were selected based on the criteria of |logFC| > 1.5 and FDR (false discovery rate) < 0.05. GO and KEGG enrichment analyses were performed using the R package clusterProfiler (4.13). Mfuzz analyses were performed using the ClusterGVis package (0.1.2) in R software (R-4.4.3). The threshold value for FDR was set at 0.05.

### 2.5 Immunofluorescence

Cells cultured from the 2.3 section were fixed with 4% paraformaldehyde for 30 min, then permeabilized in 1% Triton X-100/PBS for 20 min at room temperature and blocked with 3% BSA/PBS solution at 37°C for 1 h. Then sections were incubated with the primary antibodies (Calb1, Abcam ab229915; Vimentin, Abcam ab193555) included at 4°C overnight. After rinsing in PBS, the sections were incubated with the corresponding fluorescently labeled secondary antibody at room temperature for 1 h. Finally, the nucleus was stained with DAPI (Sigma) and inspected with a confocal laser scanning microscope (Leica, TCS SP8, Germany).

### 2.6 qPCR

Total RNA was extracted from the collected samples using Trizol, and then reverse transcription was performed with the HiScript III RT SuperMix from Vazyme to obtain cDNA. After dilution, ChamQ Universal SYBR qPCR Master Mix from Vazyme was used for qPCR, and the 2^−ΔΔCt^ method was utilized to analyze the data. The primer sequences for qPCR were listed in Table 1.

**Table 1:** Primer sequences for qPCR.

Gene	Forward	Reverse
*Rpl7*	GCAGATGTACCGCACTGAGATTC	ACCTTTGGGCTTACTCCATTGATA
*Foxa2*	TGTCAGGAGCACAAGCGAGGT	GGGTGGTTGAAGGCGTAATGGT
*Spink1*	GTGCTTTGGCCCTGCTGAGTTT	GACATCCCGCCACTGCATCATG
*Calb1*	GCAGAGTACACAGACCTCATG	GTATCCGTTGCCATCCTGATC
*Tacstd2*	GCTACTGCTACTGCTGGCGATG	TGAGCCCATTGCCCGACATTG
*Alpl*	TAACACCAACGCTCAGGTCC	TGGATGTGACCTCATTGCCC
*Osmr*	CTGGTTCCCATGGCCTCATT	CTTTCGACCAGGGGCTTCAT
*Klf4*	CCGACTAACCGTTGGCGT	CGGGTTGTTACTGCTGCAAG
*Sox9*	CACAAGAAAGACCACCCCGA	GTCTGTTCCGTGGCCTCTTC
*Greb1*	ATGGCAAGGATTCCCCCAAG	TGGCAAGATACCCAAGGCTG
*Lcn2*	GGCCAGTTCACTCTGGGAAA	TGGCGAACTGGTTGTAGTCC
*Tsc1*	CCCTCTACCTCCCCAATGGA	GAGAGCCTCCAAAGTGGGTC
*Kif5c*	ACTCTGGCAGATGTGAACGG	ACGAGAGACTTGACCTCCGA

### 2.7 Western blot

The cultured cells were collected in lysis buffer (50 mM Tris–HCl, 150 mM NaCl, 1% Triton X-100, and 0.25% sodium deoxycholate), followed by lysis on ice for 30 min. After determining the protein concentration using a BCA kit (Thermo Fisher Scientific), the samples were subjected to SDS-PAGE electrophoresis and membrane transfer. The membrane was blocked with 5% non-fat milk, incubated with anti-p-Stat3 (1:2,000, 9,145 T, Cell Signaling Technology), anti-p-Erk (1:1,000, Cell Signaling Technology, 4,370 T), then incubated with corresponding secondary antibodies labeled with horseradish peroxidase, and finally processed with an ECL Chemiluminescent kit (Amersham Biosciences) for visualization.

### 2.8 Statistical analysis

All results of the experiments were repeated at least three times independently, except for the *in vivo* study. *p*-values <0.05 were considered statistically significant. All statistical analyses were done using GraphPad Prism (GraphPad Software Inc., USA).

## 3 Results

### 3.1 Purity analysis of luminal and glandular epithelium

**Figure 2:**
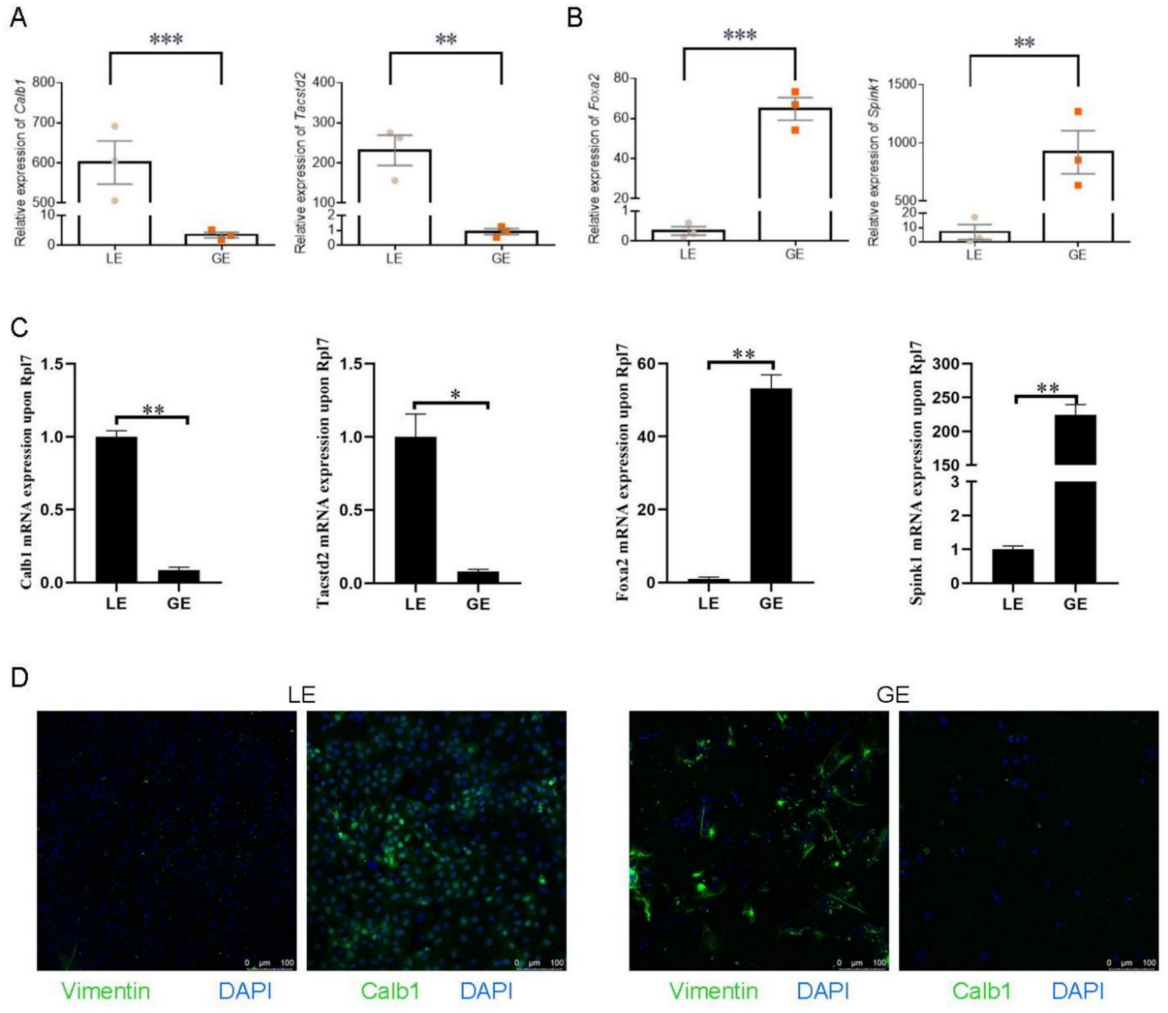
Relative expression of genes in luminal epithelium (LE) and glandular epithelium (GE). **(A)** Relative expression of *Calb1* and *Tacstd2* in LE and GE. **(B)** Relative expression of *Foxa2* and *Spink1* in LE and GE. **(C)** Quantitative PCR expression of *Calb1*, *Tacstd2*, *Foxa2*, and *Spink1*. **(D)** The fluorescence staining of Calb1 and Vimentin on LE and GE cells. Scale bar = 100 μm. Data are expressed as mean ± standard error of mean. *p < 0.05, **p < 0.01, ***p < 0.001.

The expression levels of *Calb1* and *Tacstd2* were significantly higher in the LE compared to GE (Figures 2A,C). These findings support the purity of the LE, as Calb1 and Tacstd2 are specific markers for LE (21, 22). In contrast, the markers for GE, Foxa2 and Spink1 (23, 24), were significantly higher in the GE than in the LE (Figures 2B,C), confirming the purity of the endometrial epithelial cells. Moreover, our immunofluorescence showed that LE has good quality, but there are some stromal cells in GE (Figure 2D).

### 3.2 Transcriptome analysis of different times in luminal and glandular epithelium after the mouse embryo implantation

**Figure 3:**
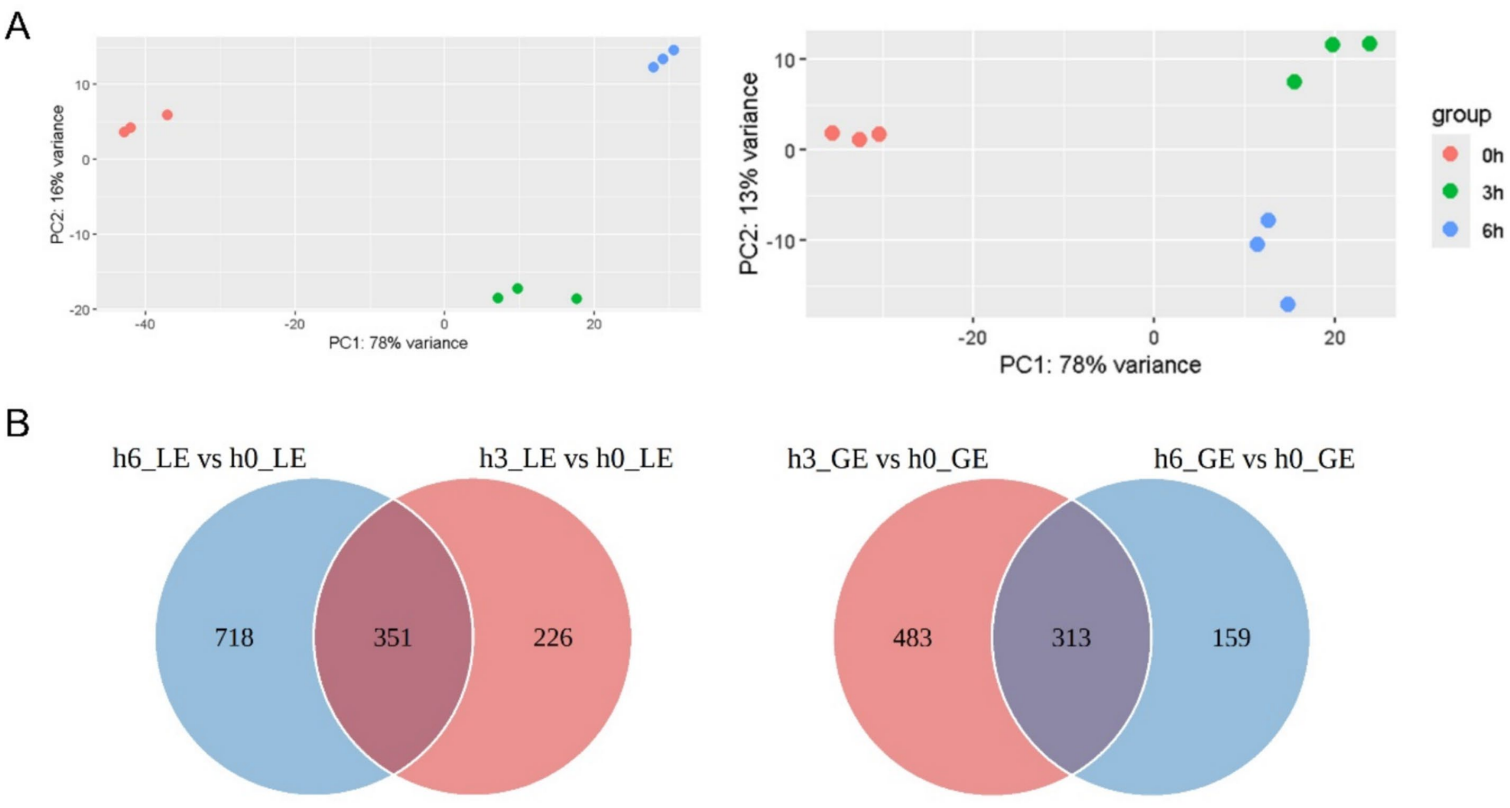
Principal component analysis (PCA) and Wayne plots of differentially expressed genes (DEGs) in luminal epithelium (LE) and glandular epithelium (GE) at different time points. **(A)** PCA plots demonstrating the sample distribution of LE and GE at 0 h (h0), 3 h (h3), and 6 h (h6) after progesterone injection. **(B)** Wayne plots demonstrating the overlap of DEGs between LE and GE at different time points.

The principal component analysis (PCA) plot showed that the distribution of samples within the same group is more concentrated, indicating that the experiments are more reproducible and that technical errors are smaller, resulting in reliable results (Figure 3A).

Differential gene expression analysis revealed that for LE, there were 1,069 differentially expressed genes between h6 and h0, and 577 differentially expressed genes between h3 and h0, resulting in a total of 351 differentially expressed genes (DEGs) for both comparisons (Figure 3B and Supplementary Table 1). For GE, there were 472 DEGs for the differentiated genes between h6 and h0, 796 DEGs for the differentiated genes between h3 and h0, and a total of 313 DEGs for both (Figure 3B and Supplementary Table 2). Functional enrichment analysis, such as Gene Ontology (GO) and Kyoto Encyclopedia of Genes and Genomes (KEGG), of overlapping genes can reveal common biological processes or pathways. We also selected some top candidate genes from RNA-seq for validation by qPCR. It showed similar results between RNA-seq and qPCR (Supplementary Figure S2).

### 3.3 Results of enrichment analysis

**Figure 4:**
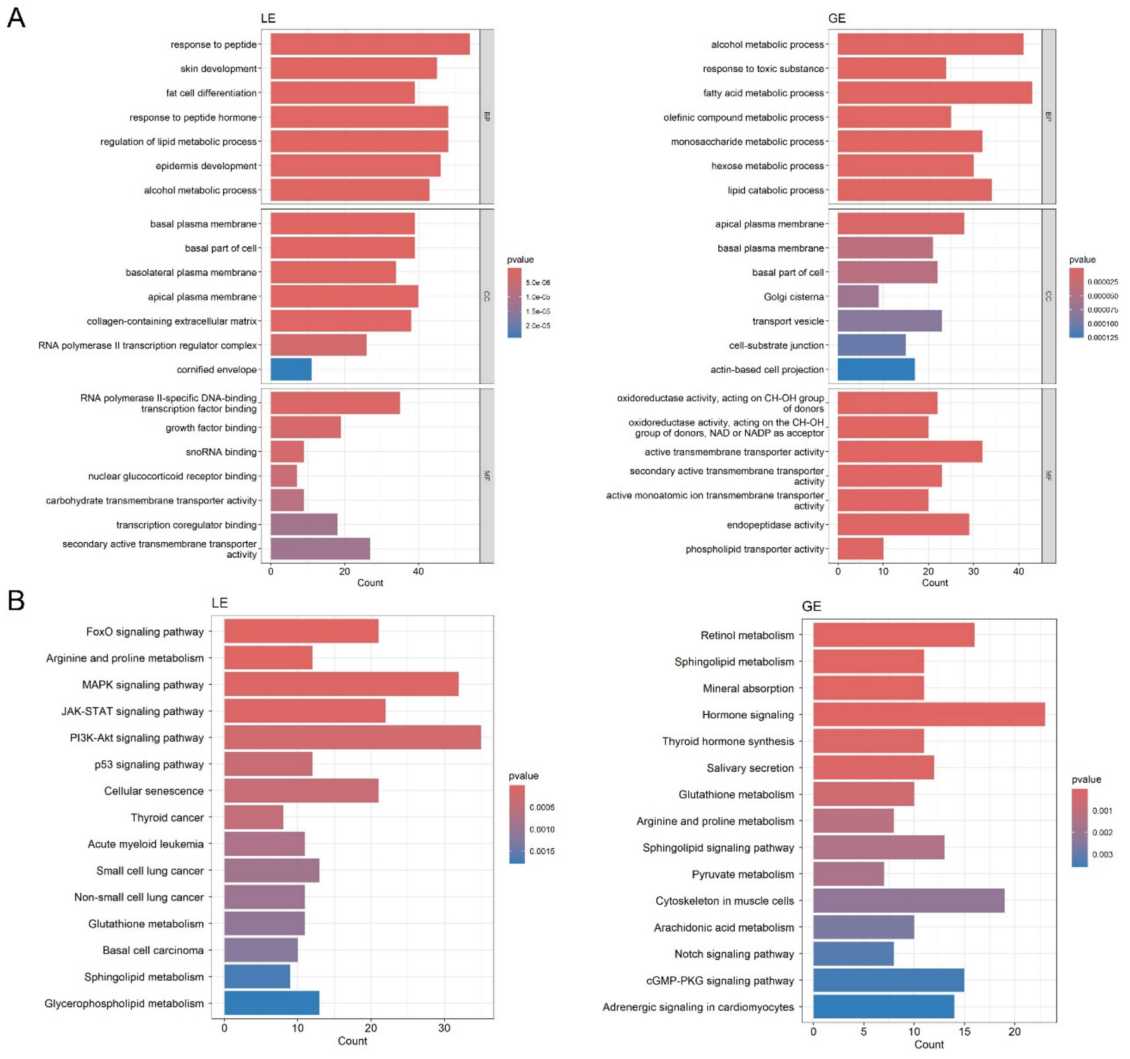
Gene ontology (GO) and Kyoto Encyclopedia of Genes and Genomes (KEGG) enrichment analysis of differentially expressed genes (DEGs) in luminal epithelium (LE) and glandular epithelium (GE). **(A)** GO enrichment analysis of overlapping DEGs from the h0–h3 and h0–h6 comparisons within each cell type. **(B)** KEGG pathway enrichment analysis of overlapping DEGs from the h0–h3 and h0–h6 comparisons within each cell type.

GO enrichment analyses of transcriptomic data from the shared differential genes in the luminal and glandular epithelia revealed distinct molecular functions and biological processes in the two epithelia during mouse embryo implantation. The enrichment analysis revealed that in the LE, significantly enriched biological processes included responses to peptides, suggesting that these processes may be involved in embryo attachment and early implantation (Figure 4A and Supplementary Table 1). In addition, significantly enriched cellular components included the basal plasma membrane and the apical plasma membrane, suggesting that the cellular polar structure of the LE plays a crucial role in embryo implantation. In terms of molecular function, RNA polymerase II-specific DNA-binding transcription factor binding and growth factor binding were significantly enriched. Suggesting LE may be involved in the early regulation of embryo implantation by modulating the activities of transcription factors and growth factors.

Biological processes that were significantly enriched in the GE included the alcohol metabolic process and the fatty acid metabolic process (Figure 4A and (Supplementary Table 2). Enriched cellular components included the apical plasma membrane, basal plasma membrane, basal part of cell, and the Golgi cisterna. In addition, oxidoreductase activity and active transmembrane transporter activity were significantly enriched in molecular function, suggesting that the GE may support embryo development by regulating redox reactions.

KEGG pathway enrichment analysis of transcriptomic data from LE and GE revealed different signaling pathways and metabolic processes in the two epithelia during mouse embryo implantation. In the LE, the significantly enriched pathways included the MAPK, JAK–STAT, and the PI3K-Akt signaling pathway (Figure 4B and Supplementary Table 1). We detected the p-Erk and p-Stat3 by Western blot. It showed p-Erk and p-Stat3 significantly increased after response to estradiol-17β stimulation in LE cells (Supplementary Figure S3). In addition, metabolic pathways significantly enriched included arginine and proline metabolism, glutathione metabolism, sphingolipid metabolism, and glycerophospholipid metabolism. In the GE, pathways significantly enriched included retinol metabolism, sphingolipid metabolism, and the notch signaling pathway (Figure 4B and Supplementary Table 2).

### 3.4 Mfuzz analysis for luminal and glandular epithelium

**Figure 5:**
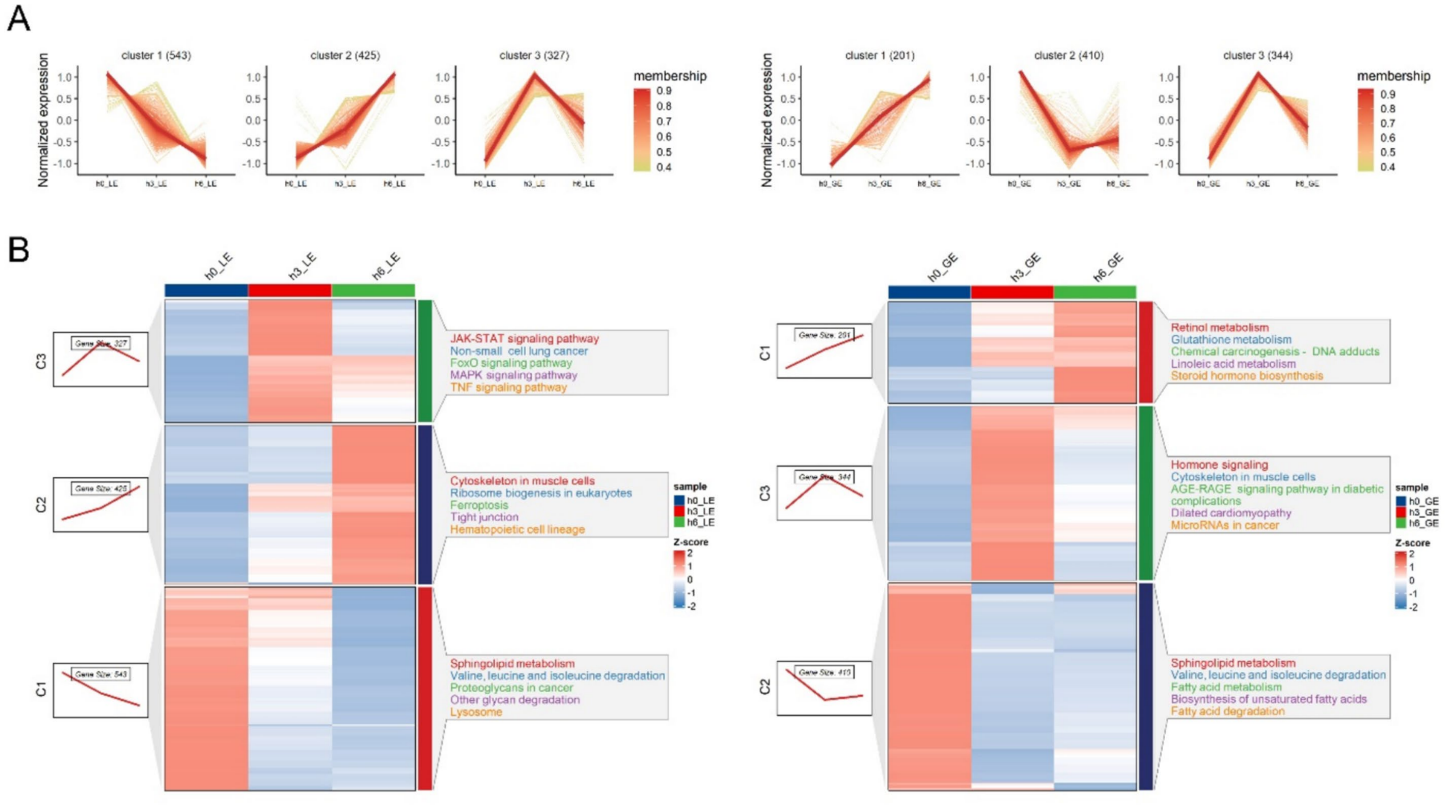
Mfuzz analysis of gene expression of luminal epithelium (LE) and glandular epithelium (GE) at different time points. **(A)** Normalized gene expression profiles of luminal epithelium (LE) and glandular epithelium (GE) at 0 h (h0), 3 h (h3), and 6 h (h6) after progesterone injection. **(B)** Clustering results of LE and GE.

Three clusters exhibiting different change trends over time were identified in LE and GE through Mfuzz analysis. For LE, the gene cluster 1 (cluster1) contains 543 genes that are downregulated from h0 to h6, cluster2 contains 425 genes that are upregulated, and cluster3 contains 327 genes that are initially upregulated at h3 to peak, followed by downregulation (Figure 5A and Supplementary Table 1). For GE, cluster 1 contains 201 genes from h0 to h6 that are upregulated. Cluster 2 contains 410 genes that are initially downregulated after h3 reaches and then slightly upregulated. Cluster 3 contains 344 genes that are initially upregulated after h3 reaches its peak and then downregulated (Figure 5A and Supplementary Table 2).

Gene expression patterns in LE and GE showed significant time-dependent changes at time points h0, h3, and h6. In LE, cluster 1 enriched pathways were sphingolipid metabolism, valine, leucine and isoleucine degradation, proteoglycans in cancer, other glycan degradation, and lysosome. These pathways are involved in metabolic processes, including lipid metabolism, amino acid metabolism, and glucose metabolism. Cluster 2 enriched pathways are cytoskeleton in muscle cells, ribosome biogenesis in eukaryotes, ferroptosis, tight junction, and hematopoietic cell lineage. JAK–STAT signaling pathway, non-small cell lung cancer, FoxO signaling pathway, MAPK signaling pathway, and TNF signaling pathway were enriched in cluster 3 (Figure 5B and Supplementary Table 1).

The GE cluster 1 enrichment pathways are retinol metabolism, glutathione metabolism, chemical carcinogenesis-DNA adducts, linoleic acid metabolism, and steroid hormone biosynthesis. Sphingolipid metabolism, valine, leucine and isoleucine degradation, fatty acid metabolism, fatty acid degradation, and biosynthesis of unsaturated fatty acids were enriched in cluster 2. Cluster 3 enrichment pathways include hormone signaling, cytoskeleton in muscle cells, AGE-RAGE signaling pathway in diabetic complications, dilated cardiomyopathy, and microRNAs in cancer. Pathways that were down-regulated in both LE and GE were sphingolipid metabolism, valine, leucine and isoleucine degradation (Figure 5B and Supplementary Table 2).

## 4 Discussion

In this study, RNA sequencing and functional enrichment analysis of LE and GE tissues collected at different time periods of activated implantation revealed the differences in function, molecular features and biological behavior between LE and GE, which provided new clues for further research on the biological functions of uterine epithelium, the mechanism of embryo implantation, as well as the diagnosis and treatment of related diseases. However, a combination of functional experiments and other molecular biology techniques is needed to verify these hypotheses.

KEGG pathway enrichment analyses in LE significantly enriched the MAPK and PI3K-Akt signaling pathways, suggesting that LE may be involved in embryo implantation by regulating these pathways. The MAPK pathway regulates cell proliferation and differentiation during embryo implantation, particularly the activation of ERK1/2 in the LE to promote embryo attachment (7, 25). Studies have shown that the MAPK pathway promotes the interaction between the embryo and the uterine epithelium by regulating the remodeling of the extracellular matrix (ECM) (26). IL-1β is a crucial mediator in regulating the implantation window during early pregnancy. It has been demonstrated that IL-1β stimulates the proliferation and development of uterine luminal epithelial cells by activating the ERK1/2 and p38 MAPK signaling pathways (27, 28). Rac1 regulates the apoptosis of uterine luminal epithelial cells through the TNFα-P38 MAPK signaling pathway, ensuring that embryos can successfully invade the uterine wall (29). The PI3K/AKT signaling pathway plays a central role in estrogen-induced *Vegfa* expression in uterine luminal epithelial cells. Estrogen activates the PI3K/AKT signaling pathway via membrane-associated ESR1 and induces *Vegfa* expression, which ultimately promotes proliferation and angiogenesis of uterine luminal epithelial cells (30). Notch signaling pathways were significantly enriched in GE in our study. The previous study demonstrated that the Notch signaling pathway plays a crucial role in the regulation of uterine GE’s proliferation, differentiation, and function, and over-activation of the Notch signaling pathway leads to hyper-proliferation of GE cells and increases their sensitivity to estrogen, which in turn affects embryo implantation (31, 32). These findings provide important clues for further investigation of the specific molecular mechanisms of LE and GE in embryo implantation.

Time-series clustering using Mfuzz revealed dynamic, compartment-specific transcriptional programs in the LE and GE in response to estradiol-17β stimulation. Notably, three distinct gene expression trajectories were identified in each epithelial subtype, highlighting temporally coordinated shifts in signaling and metabolic activity. Our results showed that the JAK–STAT and TNF signaling pathways were significantly up-regulated in the LE, indicating the JAK–STAT pathway plays an important role in embryo implantation, and these are consistent with the existing studies, activation of STAT3 through LIF, which regulates the receptivity of the uterine epithelium and embryo implantation (33). TNF-α regulates the inflammatory response, apoptosis, and immune tolerance of the endometrium through the activation of the MAPK and NF-κB pathways, which in turn affects the embryo’s attachment to the uterus (34, 35). The results also showed that sphingolipid metabolism was significantly down-regulated in GE and LE. It has been shown that sphingolipid metabolism regulates cell membrane structure and signaling during embryo implantation, particularly through sphingolipid molecules (e.g., sphingomyelin), which regulate cell proliferation and apoptosis. It is shown that this pathway plays an important role in the later stages of embryo implantation (36). Sphingolipid metabolism has been shown to play an important role in uterine epithelial receptivity and embryo attachment (37). Retinol metabolism is significantly up-regulated in the GE in our study. Retinol metabolism regulates cell differentiation and development during embryo implantation, particularly through retinoic acid (RA), which in turn regulates gene expression (38). Retinol is metabolized to produce RA, which regulates gene expression by binding to nuclear receptors, which in turn affects embryonic development and cell differentiation (39). Our time-series analysis also suggested that the valine, leucine, and isoleucine degradation pathway was decreased in the LE and GE. Future studies can further explore the effects of valine, leucine, and isoleucine degradation on embryo implantation. Furthermore, GE-specific upregulation of AGE-RAGE signaling and microRNAs in cancer suggests that GE undergoes distinct stress-related or epigenetic modulation during the receptive window, a topic warranting further investigation.

Separation of LE and GE is an important step in the study of uterine biology. Commonly used separation methods include enzymatic digestion and laser capture microdissection (LCM) (24). In this study, enzyme digestion was employed, a relatively simple and rapid technique compared to LCM, which is suitable for large-scale sample processing. However, this method has some shortcomings. Firstly, the purity of isolated glandular epithelium by this method is around 85% (22), which may contain a small number of other cell types (e.g., stromal cells or immune cells) affecting the results. Secondly, enzymatic digestion is a commonly used method for isolating luminal epithelium and glandular epithelium; however, prolonged digestion times may adversely affect cells and tissues, with subsequent effects on cell viability and function. Normal transcriptome (Bulk RNA-seq) (40), single-cell transcriptome (scRNA-seq) (41), and spatial transcriptomics (42) are three commonly used techniques for transcriptome analysis, each with its unique advantages and limitations. Only Bulk RNA-seq was used in this study. In the future, single-cell or spatial transcriptomes could be used for further studies.

In this study, the differential response mechanisms of LE and GE during embryo implantation were investigated in depth by constructing a mouse model of delayed and activated implantation. Through RNA sequencing and functional enrichment analyses of LE and GE tissues at different times after activated implantation, we found that LE might regulate embryo attachment and initial invasion through activation of JAK–STAT, MAPK, and PI3K-Akt signaling pathways. In contrast, GE may have specialized roles in supporting embryonic development and maintaining the uterine microenvironment by modulating retinol metabolism, sphingolipid metabolism, and the Notch signaling pathway. These findings provide a new perspective for an in-depth understanding of the molecular mechanism of embryo implantation, as well as an important theoretical foundation for infertility treatment and optimization of assisted reproduction techniques.

## Data Availability

The original contributions presented in the study are included in the article/Supplementary material, further inquiries can be directed to the corresponding authors.

## Supplementary material

SUPPLEMENTARY FIGURE S1 Embryo implantation in the activation mouse model.

SUPPLEMENTARY FIGURE S2 The mRNA levels of different top candidate genes.

SUPPLEMENTARY FIGURE S3 The protein levels of p-Erk and P-Stat3.
