# Correction: Different responses of luminal and glandular epithelium during mouse embryo implantation

**DOI:** 10.3389/fvets.2025.1754503

**Published:** 2026-01-08

**Authors:** Wenjing Cui, Xiangrui Guan, Peisen Liu, Qi-Xin Xu, Juan Xie, Yaqi Jiao, Lin Jin, Peng-Chao Wang, Zhenshan Yang

**Affiliations:** 1 College of Veterinary Medicine, Shanxi Agricultural University, Taigu, China; 2 Shenzhen Maternity and Child Healthcare Hospital, Southern Medical University, Shenzhen, Guangdong, China; 3 Chongqing Key Laboratory of Human Embryo Engineering and Precision Medicine, Center for Reproductive Medicine, Chongqing Health Center for Women and Children, Women and Children's Hospital of Chongqing Medical University, Chongqing, China; 4 College of Marine Life Sciences, Ocean University of China, Qingdao, China; 5 College of Veterinary Medicine, South China Agricultural University, Guangzhou, China

**Keywords:** luminal epithelium, glandular epithelium, embryo implantation, signaling pathway, RNA sequencing

In the published article, there was an error in affiliation 5.

Author Zhenshan Yang was erroneously assigned to affiliation “Department of Clinical Sciences Lund, Lund University, Lund, Sweden”. This affiliation has now been removed.

Affiliation “College of Veterinary Medicine, South China Agricultural University, Guangzhou, China” was omitted for author Zhenshan Yang. This affiliation has now been added for author Zhenshan Yang.

In the published article, Zhenshan Yang's email address was incorrect. This has been updated to: le1730@126.com.

The original article has been updated.

