# Supplementary figures and images for: Different responses of luminal and glandular epithelium during mouse embryo implantation
Source: Front Vet Sci. 2025 Sep 24;12:1661930. doi: 10.3389/fvets.2025.1661930 (PMC12506090; doi:10.3389/fvets.2025.1661930)

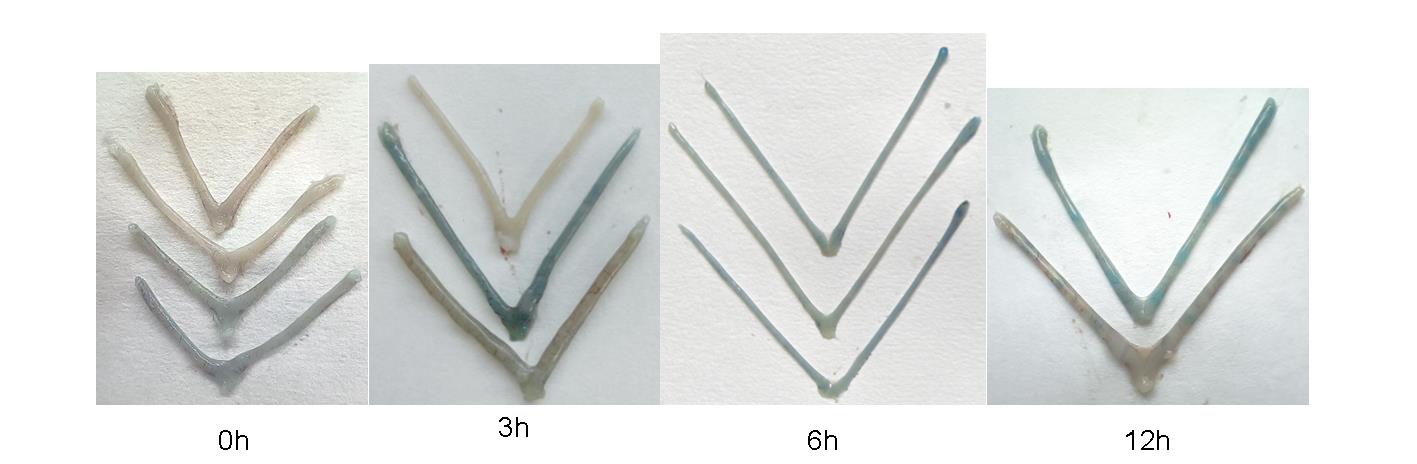

Supplement: SUPPLEMENTARY FIGURE S1 — Embryo implantation in the activation mouse model. [file Image_1.JPEG]

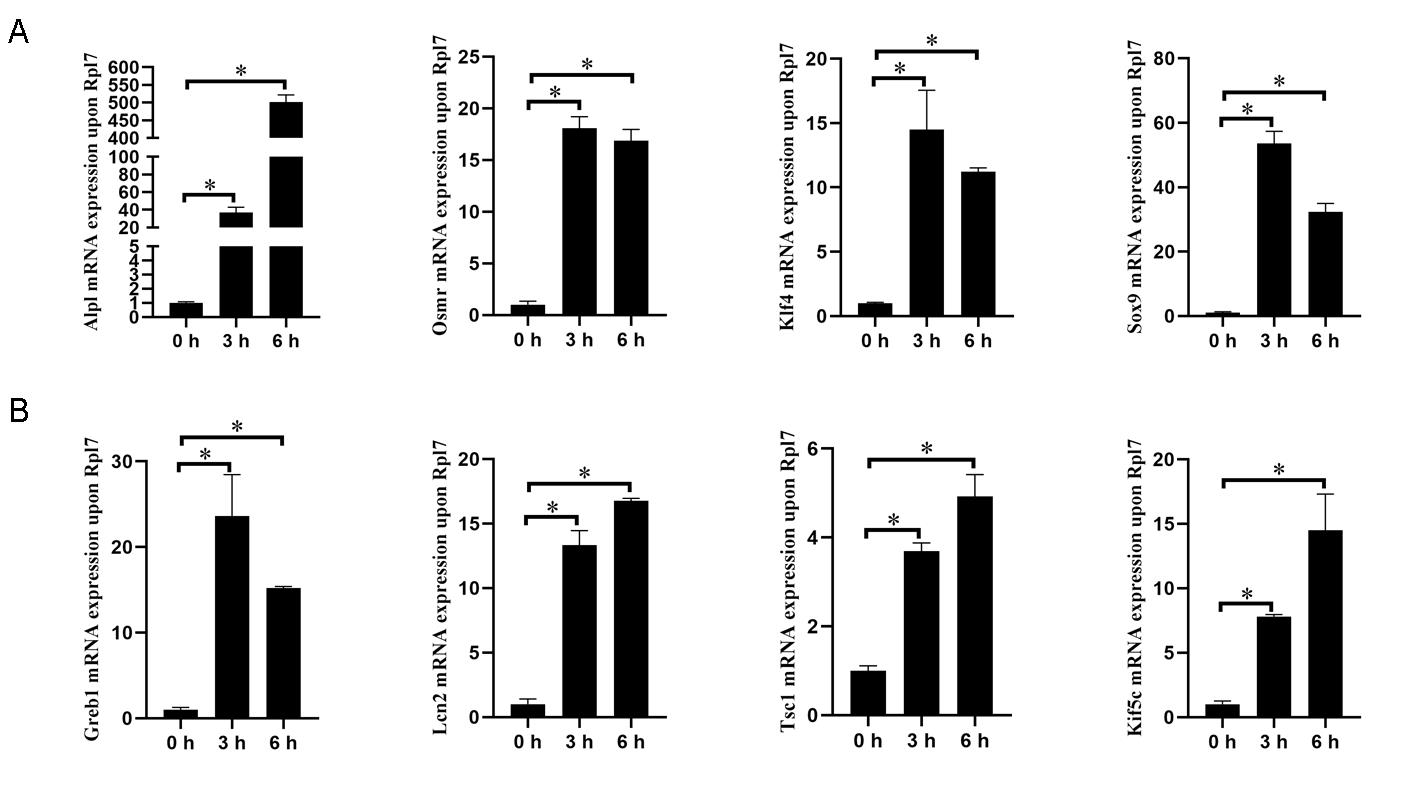

Supplement: SUPPLEMENTARY FIGURE S2 — The mRNA levels of different top candidate genes. [file Image_2.JPEG]

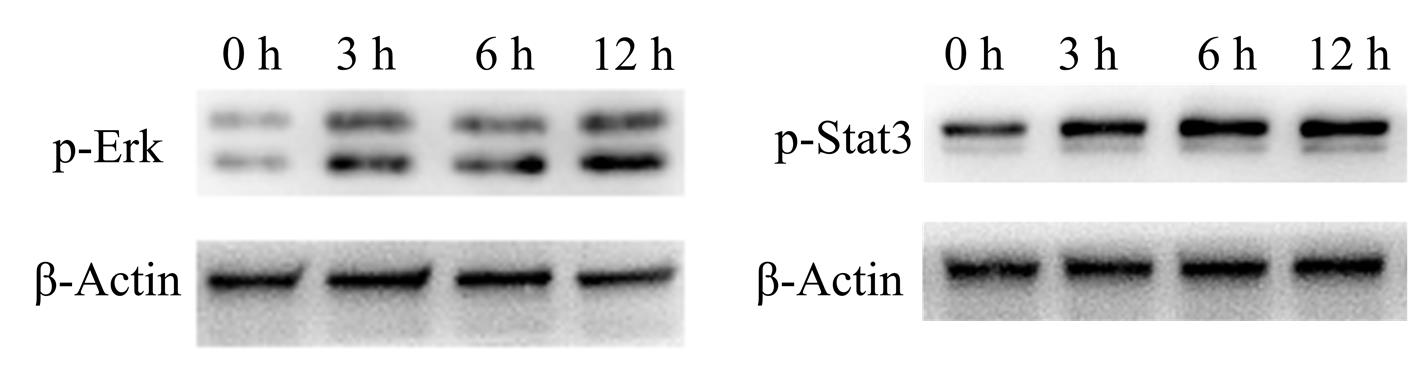

Supplement: SUPPLEMENTARY FIGURE S3 — The protein levels of p-Erk and P-Stat3. [file Image_3.JPEG]
